# The role of leptin in osteoarthritis

**DOI:** 10.1097/MD.0000000000010257

**Published:** 2018-04-06

**Authors:** Moqi Yan, Junxin Zhang, Huilin Yang, Ye Sun

**Affiliations:** a Department of Orthopedics, The First Affiliated Hospital of Soochow University; b Orthopedic Institute, Soochow University, Suzhou, China.

**Keywords:** cartilage, chronic disease, leptin, osteoarthritis, signaling pathway

## Abstract

**Background:**

The pathogenesis of osteoarthritis (OA) is not clear; leptin may be related to its pathogenesis.

**Methods:**

We reviewed articles on leptin in OA, chondrocytes, and in vitro experiments. It is concluded that leptin may lead to OA via some signaling pathways. At the same time, the concentration of leptin in vitro experiments and OA/rheumatoid arthritis (RA) patients was summarized.

**Results:**

Leptin levels in serum and synovial fluid of OA/RA patients were higher than normal person. In the condition of infection and immunity, serum leptin levels in the peripheral blood significantly increase. Because of the close relationship between obesity, leptin, and OA, it is crucial to study the effects of weight loss and exercise intervention on serum leptin levels to improve the symptoms of OA patients.

**Conclusion:**

Treatment for leptin-increased obesity may be a treatment for OA. The role of leptin in OA cannot be ignored and needs to be further studied.

## 1 Introduction

Osteoarthritis (OA) is a common degenerative disease of articular cartilage which mainly occurs in the older population.^[1]^ There are a lot of risk factors for OA, such as obesity, age, trauma, sex, and so on. And this disease will eventually lead to severe pain and joint movement disorders.^[2]^ The pathogenesis of OA has not been clear, whereas a recently found adipocyte factor called leptin was involved in the body's metabolism and the immune adjustment and its expression was significantly increased in OA patients. Leptin was considered as an important participant in the development of OA. This article will focus on the role of leptin in OA development.

## 2 Results

### 2.1 The structure and function of leptin and its relationship with OA

**Figure 1:**
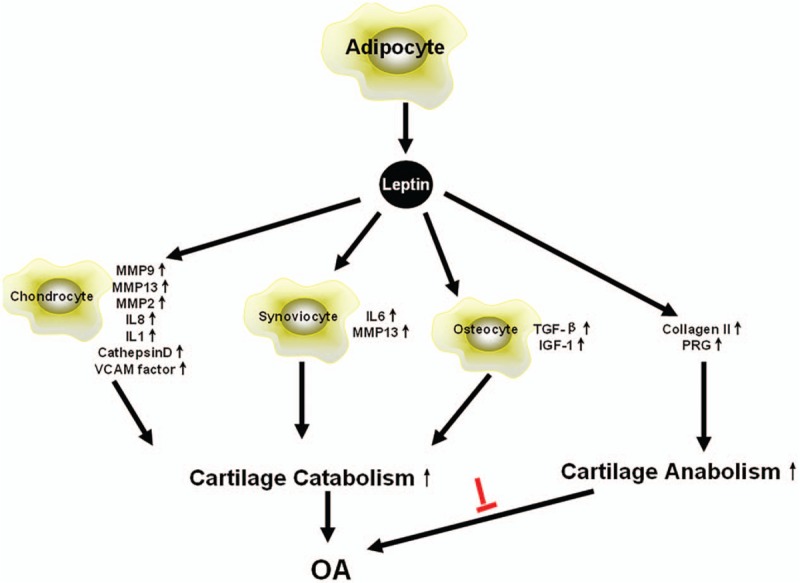
Leptin binds to the 2 CK domains and causes receptor dimerization. The dimerization receptor activates JAK2, which then phosphorylates STAT3. STAT3 forms dimers and exposes nuclear signals, then enters the nucleus to regulate gene expression.

Leptin is a peptide hormone which was first reported in 1994 mainly comes from fat tissue. This 16 kD hormone was produced by the ob/ob gene and belonged to the type 1 cytokine superfamily.^[3,4]^ Its receptor was encoded by db/db gene and belonged to the type 1 cytokine receptor superfamily.^[5]^ There are lots of homology of leptin receptors, such as obRa, obRb, obRc, obRd, obRe, and obRf,^[6]^ in which the only long receptor obRb is the most widely expression and functional receptor mainly through JAK/STAT pathway.^[7]^Figure 1 shows how JAK/STAT signaling regulates leptin expression. Like adiponectin and visfatin,^[8,9]^ leptin was also known as a adipokine,^[10]^ It was earliest found to play an important role in energy metabolism^[11]^ because it could lead to a loss of appetite and an increased energy consumption.^[12]^ Leptin levels in obesity, in turn, were significantly elevated in the human body.^[13]^ Due to its high serum levels in high weight individuals and the relieving joint symptoms by losing weight in OA patients,^[14,15]^ we hypothesized that leptin has some connections with OA caused by obesity. Afterward, leptin proved to participate in the inflammatory response which further shows that leptin may play an important role in the development of OA.^[16]^ Griffin et al proved that a lack of leptin does not cause spontaneous OA with the experiment in mouse model indicating that losing of leptin signaling pathways may protect body from the development of OA.^[17]^ There is a genetic correlation of leptin and OA.^[18]^ First, the leptin gene was increasingly expressed in OA cartilage chondrocyte. And it is also showed that leptin gene and its receptor gene are associated with OA with single nucleotide polymorphism analysis.^[19,20]^

### 2.2 The expression of leptin in serum and synovial fluid of OA patients

Normal leptin levels in human blood were related to sex and age. Argente et al found that leptin levels in female were significantly higher than male with the same age especially after the age of 12 because leptin expression decreased in male and rose in female after the age of 12.^[21]^ Leptin levels in peripheral blood of OA and rheumatoid arthritis (RA) patients were higher than normal person.^[22]^ A high aggregation phenomenon of leptin in synovial fluid of patients with RA was detected. However, its concentration was lower when compared with the serum leptin levels.^[23]^ A similar situation was found in osteoarthritic synovial fluid because the severity of OA and the level of synovial fluid leptin were positively correlated.^[24]^ Leptin levels in serum and synovial fluid of normal adults and OA/RA patients were shown in Tables 1 and 2. The expression of leptin's short receptor was detected in articular cartilage in normal people and patients with early OA, whereas the long receptor which actually involved in signaling pathways was poor expressed. In joints of patients with severe OA, the expression of leptin and its long receptor were significantly increased, and the expression of leptin and its receptor and the severity of OA were positively correlated. This suggests that the expression of leptin and its receptor increased with the development of OA.^[24,25]^

**Table 1:**
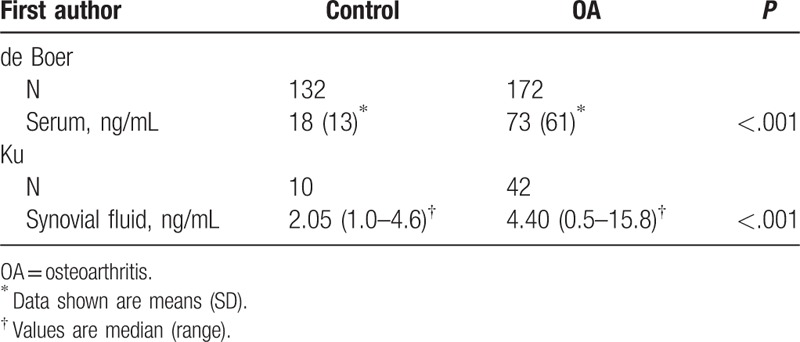
Leptin levels in serum and synovial fluid of normal adults and OA patients^[26,27]^.

**Table 2:**
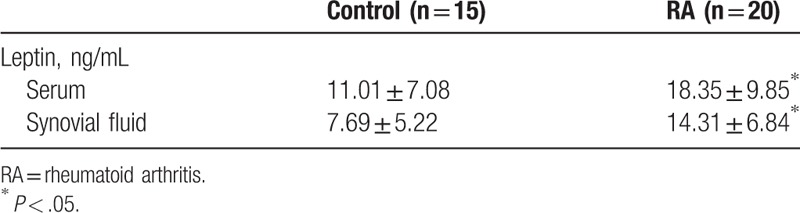
Leptin levels in serum and synovial fluid of normal adults and RA patients^[23]^.

Cartilage wear and degradation is the key step in the development of OA. The high concentration of leptin in synovial fluid and the expression of leptin receptor on cartilage cell surface indicate that leptin may act a certain role in cartilage degeneration.^[27]^ The expression of inflammation factor in normal articular cavity was scanty, whereas synovial fibroblasts presented a dose-dependent rising in the expression of IL6 when treated with leptin.^[28]^ IL6 broadly existed in serum and synovial fluid of OA patients as a kind of inflammatory cytokine,^[29]^ which can lead to degradation of proteoglycan^[30]^ and suppress the formation of cartilage by reducing cartilage proteoglycan synthesis through NOTCH pathway and increasing the expression of decomposition factor MMP13.^[31]^ The expression of IL6 decreases obviously in synovial fibroblasts in patients with OA when leptin receptor blockers were used. This suggested that leptin can induce the release of inflammatory cytokines and promote the progress of OA through cartilage damage mediated by IL6.^[28]^ Yang et al found that IL-1 expression in cartilage cells greatly increased on the seventh day when treated with leptin. In addition to IL1, content of cartilage collagen degradation-promoting factors MMP9 and MMP13 was also increased.^[25,32]^ Leptin and IL1 together could result in the abnormal expression of NOS_2_ which can promote cartilage cell apoptosis by p53 signal pathway and induce the production of matrix metalloproteinase and prostaglandin.^[28,33]^ These evidences suggested that leptin promotes the progress of OA by enhancing the NOS_2_ system with the help of IL1. In addition to the above several inflammatory cytokines, leptin also promoted the expression of other cartilage decomposition factors, such as IL8, MMP2, cathepsinD, and so on.^[34]^ What is more, leptin mediated a dose-dependent expression of VCAM in the synovium in RA and OA cartilage cells and mice ATDC-5 cells. VCAM correlated with severe OA and hand OA, and block of Leptin pathway can significantly reduce the production of VCAM.^[35,36]^

Leptin itself did not damage joints and cartilage, and its effects on cartilage were two-tier. In addition to promoting the progress of OA, leptin can also promote the synthesis of cartilage proteoglycan. Leptin expression in osteoblast was around 5 times in patients within OA patients than normal people.^[34,37,38]^ A similar phenomenon can be observed in articular cartilage cells. Osteoblast and articular cartilage cells produce increased TGF-beta and IGF-1 under leptin treatment. The 2 kinds of growth factors can promote the synthesis of proteoglycan. Leptin in low concentration can promote proteoglycan and type 2 collagen formation, whereas high concentration of leptin can induce the proliferation of articular cartilage cells.^[39]^

### 2.3 The relationship between leptin and immune-mediated OA

Leptin also participated in the immune regulating system. The expression of leptin increases under different acute inflammatory stimulation of irritants.^[40]^ In the condition of infection and immunity, serum leptin levels in the peripheral blood significantly increase.^[23]^ Interactions between Inflammatory factors will also promote the expression of leptin. For example, the level of leptin will change as the concentration of IL and TNF changes. Leptin is reflected in many autoimmune diseases, such as RA, diabetes, and multiple sclerosis.^[41]^ Pathogenesis of OA is still unclear. Recently, Wang et al found that immune responses played an important role in the onset of OA with abnormal expression and activation of complement in the synovium and synovial fluid,^[42]^ suggesting that complement was involved in the pathogenesis of OA. In addition to complement, inflammatory markers such as IL-1 beta in osteoarthritic synovial tissues were also detected,^[43]^ IL-1 beta is not only involved in the immune inflammation in the joints, but also can inhibit the type 2 collagen synthesis.^[44]^ B cells and CD4 + T cells and macrophages’ infiltration in osteoarthritic synovial tissue were also reported.^[45,46]^ All the evidence suggests the immune system was involved in the occurrence of OA. In addition to regulate metabolism, leptin also has certain effects in immune system, particularly in the T-cell proliferation and differentiation,^[47]^ and the regulation of immune function.^[48]^ Decreased T lymphocytes and immune system function were found in leptin receptor lacking mouse model.^[49]^ Leptin levels in serum and synovial fluid were increased significantly in patients with OA.^[50]^ Inflammation was weakened in leptin-lacking mouse model where symptoms of RA were also relieved. Exogenous leptin abdominal cavity injection in ob/ob mice can lead to different levels of oxidative stress and increased inflammation-related gene expression,^[51]^ indicating that leptin might change the course of the arthritis by regulating immune response in the whole body or the articular cavity.^[17]^ Synovitis is a very important process in early OA and there are now experiments targeting synovial angiogenesis early treatment to reduce symptoms of OA. Synovitis is often associated with mononuclear cell infiltrates. Leptin receptor was expressed on the surface of mononuclear cells, but whether leptin has a role in monocyte infiltration process is still not clear.^[52]^

### 2.4 The relationship between leptin, obesity, and OA

**Figure 2:**
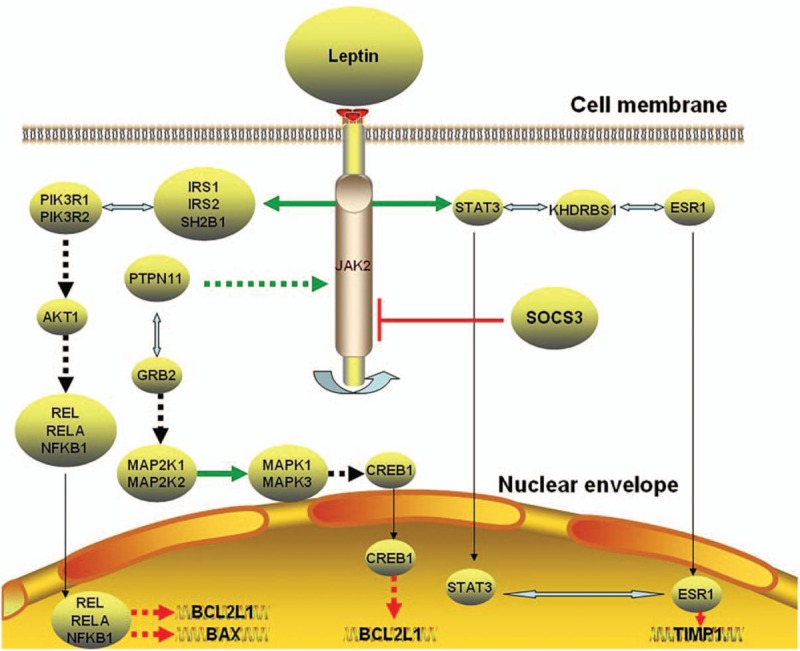
The possible role of leptin in the pathogenesis of osteoarthritis.

Obesity affects the body's material circulation and energy metabolism. Obesity is closely related to many diseases, such as diabetes, hypertension, and cardiovascular disease.^[53,54]^ OA is no exception. On the one hand, joints of obese individuals bear more pressure than the normal population, which would lead to greater joint wear.^[55]^ On the other hand, leptin levels in obese people were significantly increased may be due to leptin resistance. The increased leptin accelerated the process of inflammation of the joints.^[56]^ Recent studies have found that obese mice caused by leptin knockout were not associated with a higher risk of OA, suggesting that leptin is an essential link in obese-mediated OA.^[17]^ The possible role of leptin in the pathogenesis of OA was elaborated in Figure 2.

Leptin was mainly produced by adipose tissue in other organs, such as teeth, periodontal tissue,^[57]^ stomach,^[58]^ placenta,^[59]^ osteoblast,^[31]^ and joint cartilage.^[25]^ In addition to the synovial membrane,^[60]^ synthesis of leptin was also identified in osteoarthritic joint adipose tissue. Patellar fat pad is also a source of leptin.^[61]^ Obese individuals often present with high leptin levels because of too much fat.^[62]^ On the contrary, leptin resistance was not unusual in obese people,^[63]^ thus leading to a negative feedback between leptin sensitivity and leptin concentration and then a continuously reduction in sensitivity of leptin and an increase in leptin secretion which might explain the phenomenon that older people often present with higher OA occurrence and lower leptin sensitivity.^[64]^

Because there is such a close link between obesity, leptin, and OA, it is crucial to study the effects of weight loss and exercise intervention on serum leptin levels to improve the symptoms of OA patients. Decreases in serum leptin may be one mechanism by which weight loss improves physical function and symptoms in OA patients.^[65]^ Recent findings suggest that high-fat diet (HFD)-induced obesity can lead to the development of OA, whereas resveratrol may relieve OA pathology by reducing systemic inflammation and/or inhibiting TLR4 signaling in cartilage.^[66]^ Therefore, resveratrol may be a promising treatment for obesity-related OA. For dietary structure, dietary fatty acid content plays an important role in the pathogenesis of OA after joint injury and supports the need for further study of dietary fatty acid supplements as a potential treatment for OA.^[67]^

## 3 Conclusion

OA is a common chronic disease characterized by local inflammation, cartilage damage, great pain, and joint movement disorder. Abnormal expression of leptin was shown in patients with OA . Leptin was involved in obesity and various inflammatory processes. Its role in OA cannot be ignored. Yet the mechanism of leptin in the development of OA is still not clear. The interaction between leptin signaling pathway and other adipokines or inflammation factors and the potential immunological function of Leptin in OA still remains to be elucidated.

## Author contributions

**Conceptualization:** J. Zhang.

**Supervision:** H. Yang.

**Validation:** J. Zhang, Y. Sun.

**Writing – original draft:** M. Yan.

**Writing – review & editing:** Y. Sun.
